# Efferocytosis-induced metabolic shift in bone macrophages drives lactate production and modulates inflammation and osteoclastogenesis

**DOI:** 10.3389/fimmu.2025.1650465

**Published:** 2025-11-28

**Authors:** Rahasudha Kannan, Nicholas J. Carruthers, Amy J. Koh, Gabriel G. Kleer, Kotoba Nakamura, Stephen C. J. Parker, Laurie K. McCauley, Hernan Roca

**Affiliations:** 1 Department of Periodontics and Oral Medicine, University of Michigan School of Dentistry, Ann Arbor, MI, United States; 2 Department of Biomedical Engineering, University of Michigan, Ann Arbor, MI, United States; 3 Bioinformatics Core, University of Michigan Medical School, Ann Arbor, MI, United States; 4 Department of Computational Medicine and Bioinformatics, University of Michigan Medical School, Ann Arbor, MI, United States; 5 Department of Pathology, University of Michigan Medical School, Ann Arbor, MI, United States

**Keywords:** macrophage, efferocytosis, osteoimmunology, inflammation, energy metabolism, glycolysis, lactate

## Abstract

During the critical process of homeostatic efferocytosis, macrophages clear apoptotic cells and subsequently transition to reparative functions that promote the resolution of inflammation and support tissue repair. Their inherent plasticity enables rapid changes in macrophage activity suited to specific microenvironments. However, the heterogeneity in their cell states also presents challenges in characterizing subsets of macrophages and analyzing their specific contributions post-efferocytosis. In this study, single-cell RNA sequencing data from bone-marrow derived macrophages engulfing apoptotic osteoblasts (OB) was used to characterize macrophage subpopulations enriched during efferocytosis. Clustering analysis revealed two subpopulations (c3 and c9) that were unique to efferocytic macrophages. These distinct subpopulations displayed a transcriptional profile characterized by enhanced glycolytic energy metabolism, along with an anti-inflammatory gene signature. Notably, HIF-1 signaling, glycolysis/gluconeogenesis, and carbon metabolism were among the top five most significantly enriched pathways in c3 and c9 macrophages. qRT-PCR analysis revealed that macrophages engulfing apoptotic OBs exhibited increased expression of key glycolytic enzymes and solute carriers, including *Slc2a1, Pdk1, Ldha*, and *Slc16a3*. Metabolomics analysis revealed a significant increase in intracellular lactate, phosphoenolpyruvic acid, glycerol-3-phosphate, 2-/3-glycerophosphate, and fructose-6-phosphate, indicative of enhanced glycolysis. In addition, efferocytic macrophages showed increased extracellular lactate production compared to control macrophages, as confirmed by lactate ELISA. The effects of lactate (0-20mM) on osteoblast mineralization, osteoclast differentiation and function, and macrophage-derived inflammatory factors were evaluated through various *in vitro* experiments. While no effect was seen in osteoblast mineralization, high lactate concentrations significantly reduced the number of multinucleated osteoclasts and their resorptive activity. Interestingly, extracellular lactate also significantly upregulated M2-like macrophage markers (*Arg1, Il1rn, Klf4*). These results support the concept that macrophage efferocytosis of apoptotic osteoblasts alters macrophage energy metabolism, which in turn plays a distinct and pivotal role in modulating the bone microenvironment.

## Introduction

Efferocytosis is typically carried out by professional phagocytes such as macrophages and is specific to the engulfment of apoptotic cells. This process is crucial for development, homeostasis, repair, and regeneration (1–3). A well-known feature of homeostatic efferocytosis is its “immunological silence,” or ability to clear dead/dying cells with extreme efficiency without inducing an inflammatory response (1, 4). Though efferocytic macrophages have been shown to secrete anti-inflammatory factors (5, 6), their inherent heterogeneity and plasticity also emphasize the importance of studying cargo-specific and tissue-specific responses of efferocytic macrophages. With the majority of osteoblasts (50-70%) estimated to undergo apoptosis (7), macrophage clearance of apoptotic osteoblasts is essential for bone homeostasis (8–11) and repair (12–14). Using an inducible apoptosis model targeting osteocalcin (OCN^+^) osteoblasts, we demonstrated that induction of osteoblast apoptosis led to enhanced macrophage efferocytosis and increased trabecular bone in the vertebrae (15). While the importance of efferocytosis has been established, the specific contributions of efferocytic macrophages beyond inflammation resolution remain understudied in bone.

The engulfment of apoptotic cells is an energy intensive process, and thus, shifts in energy metabolism during efferocytosis are to be expected. Glycolysis, typically activated during periods of rapid energy demand, is most often associated with “M1-like” pro-inflammatory macrophages, whereas oxidative phosphorylation is more commonly linked to “M2-like” pro-resolving macrophages (16–19). However, emerging literature is now challenging the notion that these energy generation processes are directly or exclusively tied to macrophage inflammatory states (20). For instance, lactate, a well-known byproduct of glycolysis, has recently been shown to promote the proliferation of pro-resolving macrophages and reprogram them towards pro-reparative functions (21, 22). These mechanisms by which energy metabolism reprograms macrophages towards pro-inflammatory or pro-resolving activity remains to be elucidated.

In this study, we used single-cell RNA sequencing to identify unique subsets of efferocytic macrophages engulfing apoptotic osteoblasts and to characterize changes in their transcriptional profiles. The pronounced shift toward glycolytic energy metabolism with a simultaneous shift to an anti-inflammatory profile in these unique macrophage subsets prompted further investigation into energy metabolism in efferocytic macrophages and the effects of glycolytic metabolites on bone. Our findings suggest that the metabolic shift during efferocytosis influences not only macrophage energy generation, but also contributes to bone remodeling and pro-reparative activity.

## Materials and methods

### Animals and cell culture

All animals were maintained in accordance with institutional animal care and use guidelines, and experimental protocols were approved by the Institutional Animal Care and Use Committee of the University of Michigan. C57BL/6J (The Jackson Laboratory, Bar Harbor, ME) mice were bred in house and used for *in vitro* experiments.

Primary bone marrow cells were flushed from 4- to 6-week-old C57BL/6J mice and cultured *in vitro*. Bone marrow-derived macrophages (MΦs) were differentiated with complete α-MEM medium (10% FetalClone Serum 1 (FCS1; Cytiva Hyclone via Thermo Fisher, Waltham, MA, USA), 1x Anti-Anti (Thermo Fisher) supplemented with murine M-CSF (30 ng/mL, BioLegend, San Diego, CA, USA; 576406) for 4–6 days. Bone marrow stromal cells (BMSCs) were differentiated with α-MEM medium (20% FCSI, 1% Pen/Strep) supplemented with dexamethasone (10 nM, Sigma-Aldrich, St. Louis, MO, USA; D8893) for 6 days and used between passage 1-2.

Primary neonatal calvarial osteoblasts (OB) were collected from C57BL/6 mouse pups (< 2 weeks old). Calvaria were isolated and washed three times in 1x PBS, then plain α-MEM, followed by 3 incubations in digestion media (α-MEM, 2 mg/mL collagenase A (Roche, Pleasanton, CA, USA) or collagenase Type 2 (Worthington Biochemical, Lakewood, NJ, USA), 1x trypsin) in a 37 °C shaker (200–240 rpm) for 10 min, 10 min, and 60–90 min, with digestion media replaced between each incubation. Calvaria and the digestion media from the final digest were filtered through a 40 µm cell strainer, centrifuged (4 min, 500xg), and washed 1x in complete α-MEM before plating. Cells were expanded and used at passage 1.

### Single cell RNA sequencing experiment and analysis

**Figure 1:**
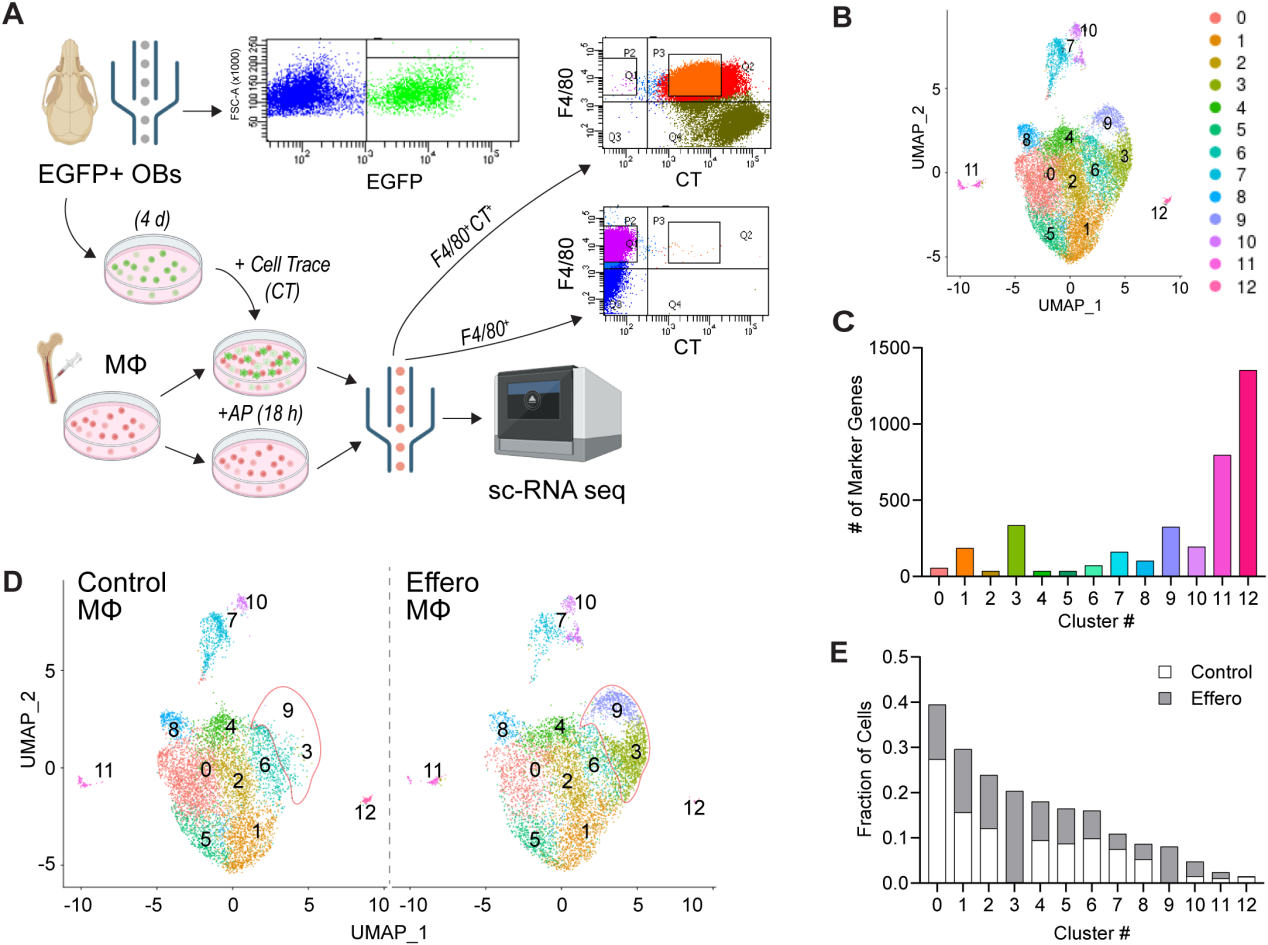
Single-cell RNA-sequencing analysis of macrophages (MΦ) engulfing apoptotic osteoblasts. **(A)** Schematic of *in vitro* efferocytosis experiment and flow cytometry gating strategy used to obtain F4/80^+^ MΦ alone (control) or F4/80^+^ MΦ engulfing Cell Trace (CT)^+^ apoptotic osteoblasts (efferocytic) samples after 18 h of co-culture for single-cell RNA-sequencing. **(B)** k-means clustering analysis of control and efferocytic MΦ, pooled together, visualized in a UMAP revealing 12 distinct clusters. **(C)** Number of marker genes identified for each cluster from analysis of pooled samples. **(D)** k-means clustering analysis, UMAPs separated by sample. Single-cell RNA sequencing analysis discriminated efferocytic MΦ from control MΦ, and identified two unique efferocytic MΦ clusters, c3 & c9, marked in red outline. **(E)** Fraction of the number of control or efferocytic MΦ in each cluster divided by total respective sample (e.g. fraction of control MΦ in c1 = # of control MΦ in c1 divided by total # of control MΦ).

Primary calvarial osteoblasts were collected (as described above) from OSXCre-iCasp9 (OSXCre^+/–^iCapase9^flox/flox^) mice, in which approximately 20% of the cell population are OSX^+^ (GFP^+^) (**Figure 1A**). Briefly, these mice express Cre recombinase under the control of the Osterix (OSX) promoter, allowing for recombination of a floxed inducible caspase-9 (iCasp9) cassette. Upon treatment with the dimerizer AP20187 (AP), OSX^+^ cells undergo apoptosis, either *in vitro* or *in vivo* (15, 23). Collected cells were cultured, then stained with CellTracer CFSE (CT^+^; ThermoFisher) prior to use. Macrophages were then co-cultured with the CT^+^ osteoblasts and treated with AP overnight. Control macrophages were cultured alone and treated with vehicle overnight. All samples were then stained with F4/80-APC (ThermoFisher) and flow cytometric sorting was performed. F4/80^+^(APC)/CT^+^ (efferocytic) macrophages and control F4/80-APC^+^ (non-engulfing) macrophages were sorted, and each cell population was subsequently processed for single-cell RNA sequencing (**Figure 1A**). Briefly, cells were combined with an RT-mix and up to eight samples loaded in wells of a Chromium chip. Partitioning oil and beads (each containing a polydT sequence, a 10x barcode sequence, and a unique molecular identifier sequence for each barcode), were also loaded on the chip. The 10x Chromium machine captures a single barcoded bead in an oil droplet, along with zero, one, or two cells according to a Poisson distribution, such that 60% of the loaded cells are captured. Following capture in the oil droplet, the mRNA was reverse transcribed into cDNA, and a portion was then amplified by PCR and fragmented. Adaptors were ligated and the product amplified again, using one of 96 indexing primer sets. Invitrogen Dynabeads and Beckman Coulter SPRI select beads were used to purify the product and ensure the final size range of the library to be sequenced was 200-800bp.

### Bioinformatics analysis

RNA-seq reads were aligned with STARsolo (v2.7.10) (24) and background subtraction and cell detection were done using cellbender (v0.3.0) (25). Droplets were accepted if they had < 6% mitochondrial content and > 600 UMI and a 99% probability of being non-empty according to cellbender. Doublets were identified with DoubletFinder (v2.0.3) (26) using a classification neighborhood coefficient (pK) of 0.17. Cells flagged as doublets by DoubletFinder were removed. After filtering for good quality cells, 7301 remained for Sample 1 (control macrophages) and 6964 for Sample 2 (efferocytic macrophages). Data from the two samples were integrated and submitted together to clustering, dimension reduction analyses and marker gene identification using Seurat (v4.0.6) (27). The “FindMarkers” function using the Wilcoxon Rank Sum Test was used for cluster marker identification. Genes had to be expressed in at least 10% of cells in a cluster to be submitted for testing and have an adjusted p-value less than 0.05 and absolute linear fold change greater than 1.5 for cells in a cluster relative to all other cells to be considered a marker. Markers were submitted to iPathway Guide for functional enrichment testing. Pathway enrichment was evaluated using Impact Analysis (28) and Gene Ontology category enrichment was evaluated using a hypergeometric test.

To identify a progression of cell states on the UMAP throughout the clustering, pseudotime analysis was performed using Monocle3 (29). The graph node that was the closest node to most cells in cluster 0 was chosen as the starting node for the pseudotime analysis. Next, a single trajectory branch that spanned cluster 0 to cluster 9 was selected for further analysis. All cells whose nearest nodes were in that branch were selected. Genes in those cells were tested for a significant relationship to cell pseudotime using Moran’s I test. To reduce noise due to dropout in individual cells, cells were pooled according to their closest node in the trajectory and median expression and pseudotime values were used. The top 1000 genes whose expression shows the strongest association (lowest p-value) with pseudotime were normalized to be between 0 and 1, grouped into 5 clusters (referred to as gene profiles later in the manuscript) using k-means clustering and submitted to iPathway guide for functional enrichment analysis.

### Efferocytosis assay

Macrophages (MΦ) were plated at 1x10^6^ cells/well in 6-well plates for qRT-PCR, metabolomics, and ELISA the day before experiment. OB (cultured to confluency) were rinsed with 1x PBS and exposed to UV light (Fisher, Transilluminator F8TIV-816) in 1x PBS for 30 min to induce apoptosis. Apoptotic OB cells (AC) were then incubated in growth media for 1 h at 37 °C, harvested using a cell scraper, filtered through a 40 µm cell strainer, and counted via trypan blue exclusion (to confirm cell death). For FACS experiments, AC were stained with Cell Tracker CFSE. AC were added to MΦ cultures at a 2:1 AC to MΦ ratio for 18 h in low-serum media (1% FCS1, 1x Anti-Anti (Antibiotic-Antimycotic), 30 ng/mL murine M-CSF). Samples were then prepared for qRT-PCR, metabolomics, or extracellular lactate assays.

### qRT-PCR

Total RNA was isolated from macrophages using Qiagen (Germantown, MD, USA) RNeasy Mini Kit. Reverse transcription was conducted using the High-Capacity cDNA Reverse Transcription Kit (Thermo Fisher, 4368814), and cDNA products were amplified and detected using TaqMan Gene Expression Master Mix (Thermo Fisher, 4369016) and TaqMan probes listed in **Table 1**. Quantitative Real time PCR was analyzed on QuantStudio 3 (Applied Biosystems via Thermo Fisher).

**Table 1:** List of TaqMan probes used for qRT-PCR analysis.

Gene	TaqMan Probe ID
*Slc2a1*	Mm01275814_m1
*Hk2*	Mm00443385_m1
*Pdk1*	Mm00554300_m1
*Ldha*	Mm01612132_m1
*Slc16a3*	Mm00446102_m1
*Il1b*	Mm00434228_m1
*Tnf*	Mm00446003_m1
*Arg1*	Mm00469812_m1
*Il1rn*	Mm00446186_m1
*Klf4*	Mm00516014_m1
*Mrc1*	Mm01329359_m1
*Il10*	Mm01288386_m1
*Atf4*	Mm00515325_g1
*Jun*	Mm07296811_s1
*Nos2*	Mm00440502_m1

### Metabolomics analysis

Macrophages were prepared per instructions from University of Michigan Metabolomics Core. In brief, cells were quickly washed with 150 mM ammonium acetate (Sigma-Aldrich, A7330), quenched with liquid nitrogen, placed on dry ice, and submitted to the Metabolomics Core for Glycolyis-TCA metabolite analysis. All analytes and Internal Standards were measured on an accurate QTOF mass spectrometer.

### Extracellular lactate quantification

Extracellular lactate in conditioned media was quantified using the Sigma-Aldrich^®^ Lactate Assay Kit (Sigma-Aldrich, MAK064). Macrophage conditioned media was collected from MΦ alone, AC alone, and MΦ+AC 18 h co-culture.

### Mineralization assay

Primary calvarial osteoblasts (OBs) and primary bone marrow stromal cells (BMSCs) were plated in 12-well plates at 300k and 800k cells/well, respectively. Cells were switched from their respective growth media to osteogenic media (10% FCSI, 1x Anti-Anti, 10 mM β-glycerophosphate (Sigma), and 50 µg/ml ascorbic acid (ThermoFisher)) with lactate (0, 10, or 20 mM; Sigma, 71718). Osteogenic media with lactate was replaced every 2–3 days for 2 weeks (BMSCs) or 3 weeks (OBs). Mineralization was visualized via von Kossa staining. Brightfield images were taken on the Leica THUNDER imaging system (Leica Microsystem, ZQQ, DE) and quantified using ImageJ (30).

### Osteoclast differentiation and functional assays

Macrophages were plated at 100,000 cells/well in 96-well plates and incubated in osteoclast differentiation media (10% FCSI, 1x Anti-Anti, 30 ng/mL murine MCSF, 100 ng/mL RANKL (Peprotech via ThermoFisher; 315-11) with lactate (0, 2.5, 5, or 10 mM). Osteoclast differentiation media was replaced every 2 days for 5–7 days, until multi-nucleated osteoclast formation was observed. Osteoclasts were visualized via TRAP staining (Leukocyte Acid Phosphatase (TRAP) Kit, Sigma, 387A). Brightfield images were taken on the Leica THUNDER imaging system and multi-nucleated (≥ 3 nuclei) cells were enumerated via ImageJ. To determine lactate effects on osteoclast function, 96-well plates were calcium coated following a Simple Pit Assay Protocol to visualize and quantify osteoclastic resorption *in vitro* (31). Macrophages were plated on the calcium coated plate at 100,000/well with osteoclast differentiation media plus lactate (0 or 10 mM). Media was replaced every 2–3 days for 9 days. Cells were fixed with 4% paraformaldehyde and imaged on the Leica THUNDER imaging system. Percent of resorption area was determined using ImageJ.

### Macrophage co-culture with lactate

Macrophages were plated at 1x10^6^ cells/mL (2 mL total volume) into 6-well tissue culture plates, and allowed to adhere for 2–3 h. For treatment, MΦs were switched to low serum media (1% FCSI, 1x Anti-Anti, no MCSF) and treated with 20 mM lactate in culture media. After 16 hours of incubation, RNA was harvested for subsequent qPCR analysis.

### Statistics

Statistical analyses were performed using GraphPad Prism 10 (GraphPad Software, version 10.2.3, San Diego, CA, USA) or using Advaita Software analysis with Bonferroni correction as specified in figure legends; p < 0.05 considered significant.

## Results

### Macrophage subsets unique to efferocytosis identified via single-cell RNA sequencing

Efferocytosis is known to modulate macrophage activity, typically promoting an anti-inflammatory and pro-reparative phenotype. Identifying the changes in macrophage profiles and the underlying mechanisms driving the reprogramming is essential to understanding the specific contributions of efferocytosis to homeostasis and tissue repair. To characterize changes in macrophage subsets as a function of efferocytosis, single-cell RNA sequencing (scRNA-seq) was performed using the 10X Genomics platform on flow-sorted control macrophages (F4/80-APC^+^) and efferocytic macrophages engulfing Cell-Trace-stained apoptotic osteoblasts (F4/80-APC^+^CT^+^) (**Figure 1A**). Sequencing data for both samples were integrated prior to applying unsupervised nearest neighbor clustering analysis. Visualization using UMAP revealed 12 macrophage clusters (**Figure 1B**). Clusters 12, 11, 9, and 3 exhibited the highest number of marker genes that distinguished them from other clusters (**Figure 1C**), although clusters 11 and 12 also contained the fewest cells (**Figures 1D, E**).

When control and efferocytic macrophages from the common UMAP and cluster analysis were plotted separately, clusters 3 and 9 (c3 and c9) were found to be unique to efferocytic macrophages (**Figure 1D**). Quantification of the proportion of control and efferocytic macrophages within each cluster confirmed that c3 and c9 were predominantly composed of efferocytic macrophages (**Figure 1E**). In contrast, cluster 0 (c0) was composed of noticeably fewer (< 50%) efferocytic macrophages than control macrophages. The distinct shifts in clusters 0, 3, and 9 suggest that cluster 0 macrophages may transition toward cluster 3 and 9 macrophages during efferocytosis.

### Unique efferocytic macrophages have a glycolytic metabolism and anti-inflammatory profile

**Figure 2:**
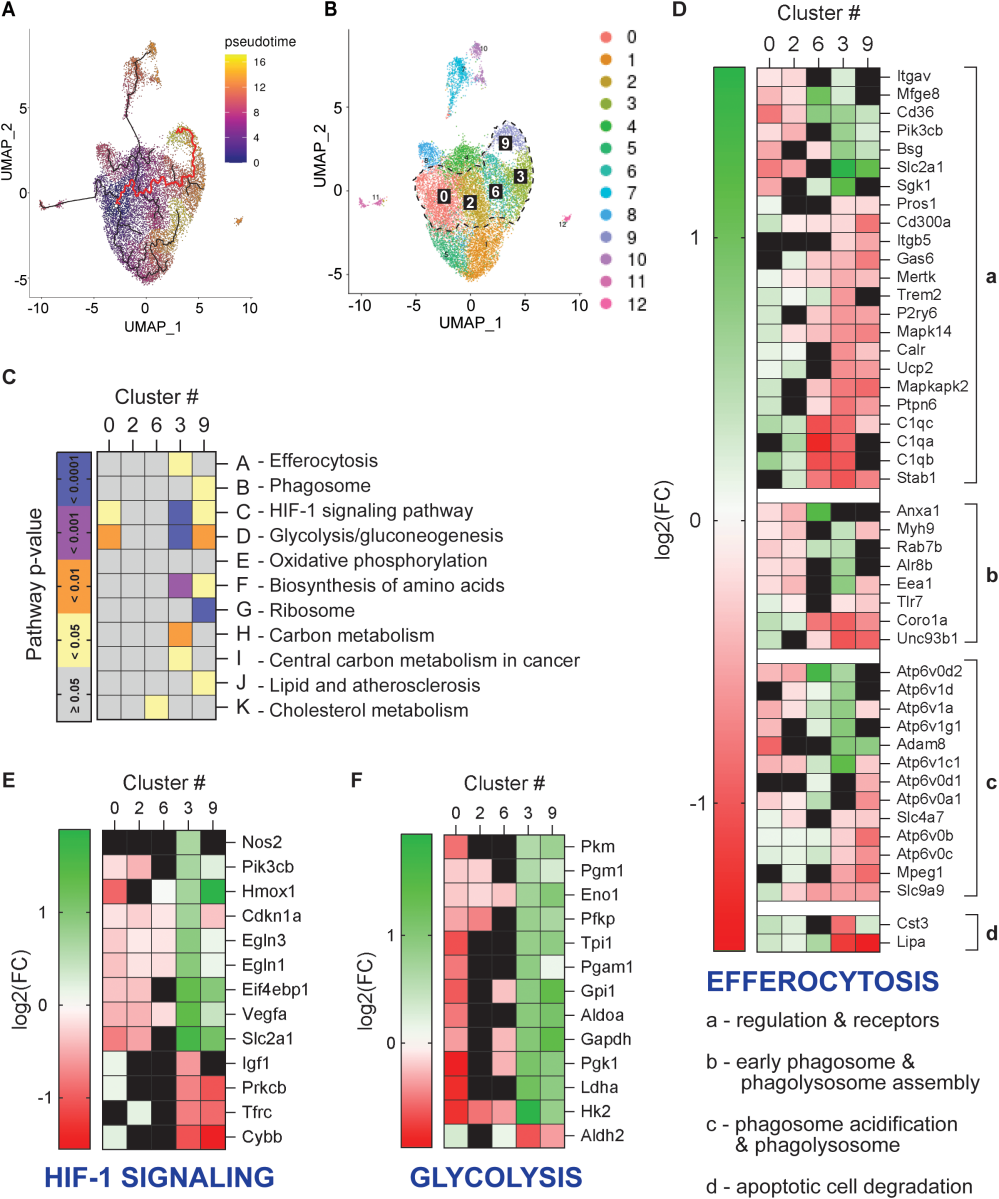
Characterization of efferocytosis & energy metabolism in MΦ along pseudotime trajectory to unique efferocytic clusters. Single-cell RNA-sequencing and k-means clustering analysis were performed on pooled control and efferocytic MΦ samples from *in vitro* efferocytosis experiment described in **Figure 1**. The pooled, clustered MΦs underwent pseudotime trajectory analysis with c0 as the selected starting point. **(A)** Pseudotime trajectory analysis revealed several pseudotime trajectories, with the trajectory of interest (from c0 to unique clusters c3 and c9) highlighted in red. **(B)** UMAP shows the trajectory of interest (red line from **(A)**) travels from c0, through c2 & c6, to c3 & c9. **(C)** Heatmap of selected pathways related to phagocytosis (A, B) and energy metabolism (C–K) for clusters along trajectory of interest. Pathways not expressed or with adjusted p > 0.05 (Impact Analysis) are shown in black. **(D–F)** Heatmaps of curated genes in **(D)** efferocytosis, **(E)** HIF-1 signaling, and **(F)** glycolysis for clusters along trajectory of interest. Genes not expressed or with adjusted p > 0.05 (Wilcoxon Rank Sum Test) are shown in black.

Pseudotime trajectory analysis applied to the combined macrophage samples identified several trajectories originating from cluster 0, but only one progressed toward the unique subsets, c3 and c9 (**Figure 2A**). This distinct pseudotime trajectory, overlaid on the UMAP clusters, revealed the transition of macrophages along the path (c0 → c2 → c6 → c3 → c9) towards the unique efferocytic macrophage populations (**Figure 2B**). Pathway analysis via the Advaita iPathwayGuide software was performed on marker genes identified for each macrophage cluster to detect their distinguishing features and functions. Among the top pathways enriched in c3 and c9 were several pathways related to energy metabolism, prompting a broader evaluation of metabolic pathways (**Supplementary Figure 1**). Other clusters were neither enriched in as many metabolic pathways, nor exhibiting the same level of pathway significance as c3 and c9 (**Supplementary Figure 2A**). Other functions of interest such as cell cycling, replication, antigen presentation, and bone remodeling were not denoted as significant pathways in c3 and c9 (**Supplementary Figure 2A**).

A curated list of pathways relevant to efferocytosis and energy metabolism confirmed phagocytic processes and strong shifts in energy metabolism in c3 and c9 (**Figure 2C**). Interestingly, HIF-1 signaling and glycolysis were significant in clusters 0, 3, and 9, while the oxidative phosphorylation pathway was not. To characterize the directionality of changes in efferocytosis, HIF-1 signaling, and glycolytic metabolism, genes relevant to these pathways were visualized in heat maps (**Figures 2D–F**). As all twelve subsets in the clustering analysis contained efferocytic macrophages (**Figure 1E**), efferocytic gene expression also visualized across all clusters (**Supplementary Figure 2B**). While it is not possible to determine the exact stage of efferocytosis represented in each cluster, many efferocytosis receptors and phagolysosome-associated genes were upregulated in cluster 3, confirming this macrophage subset was actively undergoing efferocytosis (**Figure 2D**). The majority of HIF-1 signaling and glycolysis-associated genes clearly transitioned from downregulation in cluster 0 to upregulation in clusters 3 and 9, suggesting enhanced glycolytic metabolism in efferocytic macrophages (**Figures 2E, F**). While many oxidative phosphorylation related genes were also regulated oppositely in c0 vs. c3 and c9, a clear pattern in activation or deactivation of this metabolic pathway was not apparent (**Supplementary Figure 2C**).

**Figure 3:**
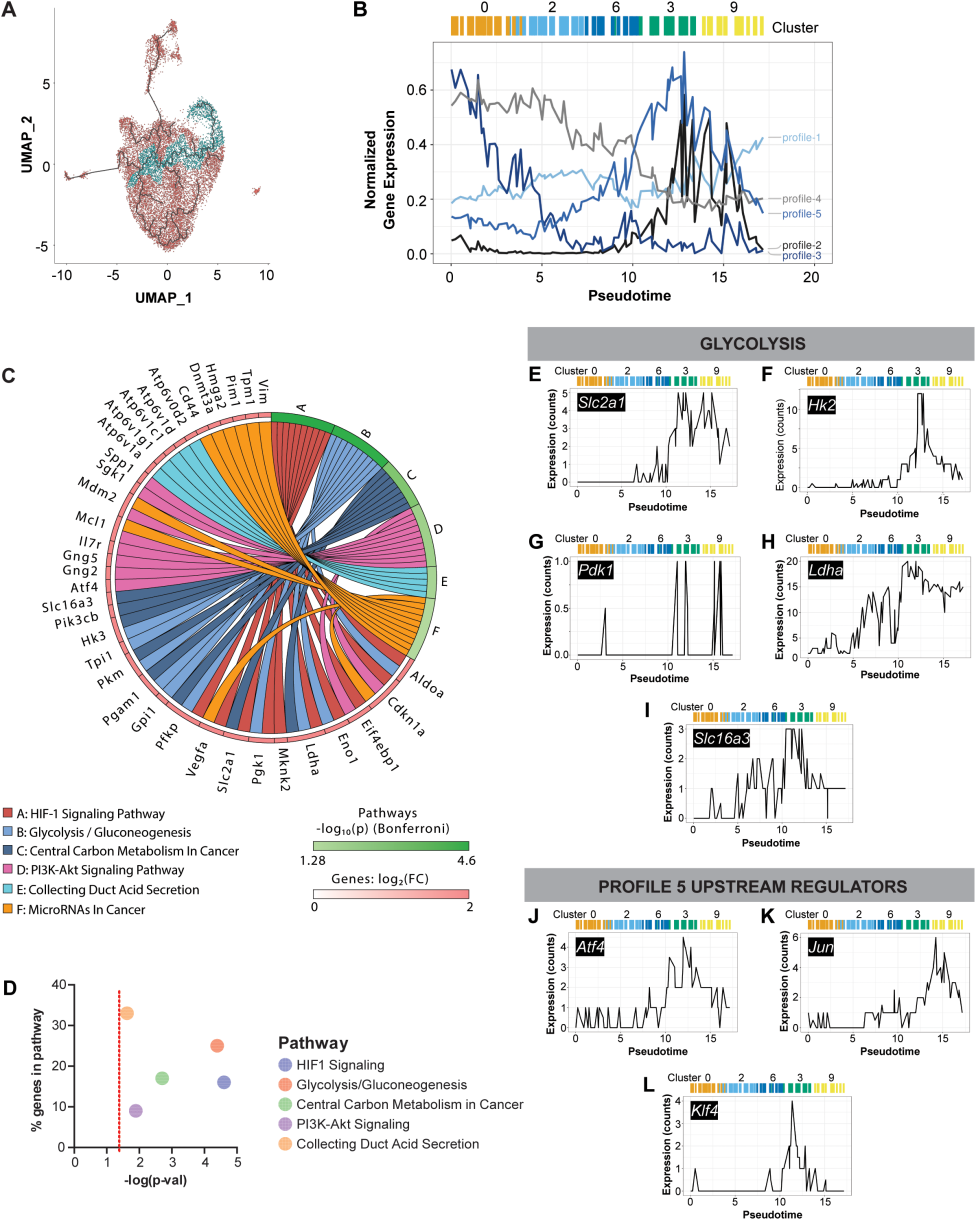
Gene profile analysis of pseudotime trajectory to unique efferocytic clusters. The pseudotime trajectory of interest identified in **Figure 2A** was analyzed separately to investigate gene expression profiles along this trajectory. Analysis was performed on the previously pooled and clustered MΦ single-cell RNA-sequencing data. **(A)** Pseudotime trajectory of interest was superimposed on UMAP and included cells closest to the trajectory (indicated in blue) while all other cells are shown in red. These cells were used to identify top 1000 genes with a significant relationship to pseudotime. These genes were clustered into five groups based on their expression profile along the trajectory. **(B)** Line plot depicting the five profiles of gene expression identified along trajectory of interest. Relative position of clusters along this trajectory (c0, c2, c6, c3, c9) and pseudotime are depicted to highlight transitions observed from c0 to c3 and c9. **(C)** Summary plot from Advaita iPathwayGuide for gene profile #5. Top pathways identified for profile 5 included HIF-1 signaling, glycolysis, and central carbon metabolism. Statistical significance evaluated using Bonferroni correction, –log(p) > 1.3 (equivalent to p < 0.05, Impact Analysis) was considered significant. **(D)** Dot plot of significance vs. % genes in the top pathways identified for profile 5. The dotted red line indicates the significance threshold, -log(p) = 1.3. **(E–L)** Plots of gene expression along trajectory of interest and pseudotime for individual genes involved in glycolysis **(E–I)** and upstream regulation of genes in profile #5 **(J–L)**.

Initial analysis of gene expression in clusters along the trajectory toward the unique efferocytic macrophage subsets (c0, c2, c6, c3, c9) revealed a clear shift in energy metabolism toward glycolysis. However, many cells within these clusters appeared spatially distant from the trajectory of interest. Thus, we performed additional analyses focused exclusively on cells nearest to the trajectory (**Figure 3A**). These selected macrophages were examined for genes significantly associated with pseudotime, identifying those with distinct expression patterns along this trajectory. The top 1000 genes were subjected to k-means clustering, resulting in five distinct gene expression profiles (**Figure 3B**). Pathway analysis using Advaita iPathwayGuide identified the top pathways associated with each gene profile (**Figures 3C, D**; **Supplementary Figure 3**). Notably, profile 5 included pathways enriched in c3 and c9, such as HIF-1 signaling, glycolysis, and central carbon metabolism (**Figures 3C, D**). As this focused analysis also pointed to shifts in energy metabolism, the expression of select glycolytic genes along the trajectory were plotted (**Figures 3E–I**). All five genes shown (*Slc2a1, Hk1, Pdk1, Ldha, Slc16a3)* had an increased magnitude of expression as they approached c3 and c9, often peaking in c3. Next, we used the “upstream regulators” features in Advaita iPathwayGuide to identify factors potentially driving the shift in macrophage energy metabolism, focusing on profile 5. This analysis revealed three transcription factors—*Atf4, Jun, and Klf4*—all of which exhibited increased expression along the pseudotime trajectory, peaking in cluster 3 (**Figures 3J–L**).

**Figure 4:**
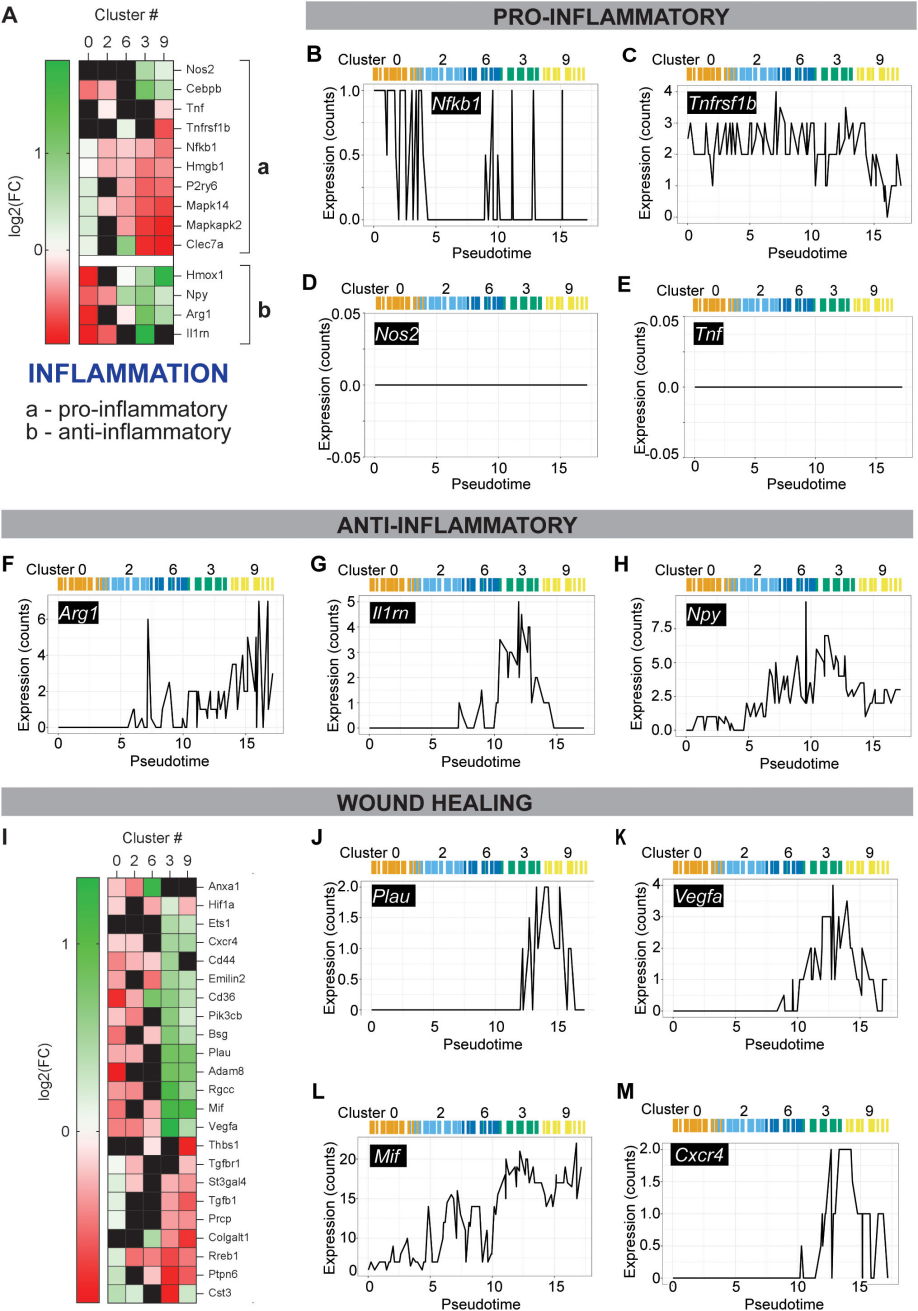
Characterization of inflammation and wound healing in MΦs along pseudotime trajectory to unique efferocytic clusters. **(A, I)** Heatmaps of curated genes involved in **(A)** pro- or anti-inflammatory activity, and **(I)** wound healing (angiogenesis, ECM remodeling, and inflammation) shown for clusters along trajectory of interest (c0, c2, c6, c3, c9). Genes not expressed or with adjusted p > 0.05 (Wilcoxon Rank Sum Test) in heatmaps are shown in black. **(B–H, J–M)** Plots of gene expression along trajectory of interest and pseudotime for selected genes involved in **(B–E)** pro-inflammatory activity, **(F–H)** anti-inflammatory activity, and **(J–M)** wound healing. Relative position of clusters along this trajectory (c0, c2, c6, c3, c9) and pseudotime are depicted to highlight transitions observed in c3 and c9.

Lastly, the inflammatory and wound healing profiles of macrophages along the trajectory toward distinct efferocytic subsets were evaluated using both approaches: gene expression in clusters along the trajectory, and expression in cells nearest to the trajectory. A clear transition toward an anti-inflammatory profile, characterized by both the suppression of pro-inflammatory genes and the upregulation of anti-inflammatory genes, was observed in c3 and c9 (**Figure 4A**). Some pro-inflammatory genes, such as *Nfkb1* and *Tnfrsf1b* (**Figures 4B, C**), showed reduced frequency or magnitude of expression as cells approached c3 or c9, while others, such as *Nos2* and *Tnf* (**Figures 4D, E**), were not expressed at all along the trajectory. In contrast, anti-inflammatory genes including *Arg1, Il1rn*, and *Npy* exhibited increased expression approaching c3 (**Figures 4F–H**). Genes associated with angiogenesis, extracellular matrix (ECM) remodeling, and inflammation regulation, collectively grouped under “wound healing”, displayed an opposing pattern of regulation in c0 vs. c3 and c9 (**Figure 4I**). Selected wound healing genes, including *Plau, Vegfa, Mif*, and *Cxcr4*, showed expression profiles similar to the glycolytic and anti-inflammatory genes, with upregulation as they approach cluster c3 (**Figures 4J–M**). Expression of inflammation- and wound healing-associated genes was also visualized across all clusters (**Supplementary Figure 4**), with other clusters showing neither enrichment in these pathways nor the same level of significance as c3 and c9.

### Macrophages shift to glycolytic energy metabolism during efferocytosis of osteoblasts

**Figure 5:**
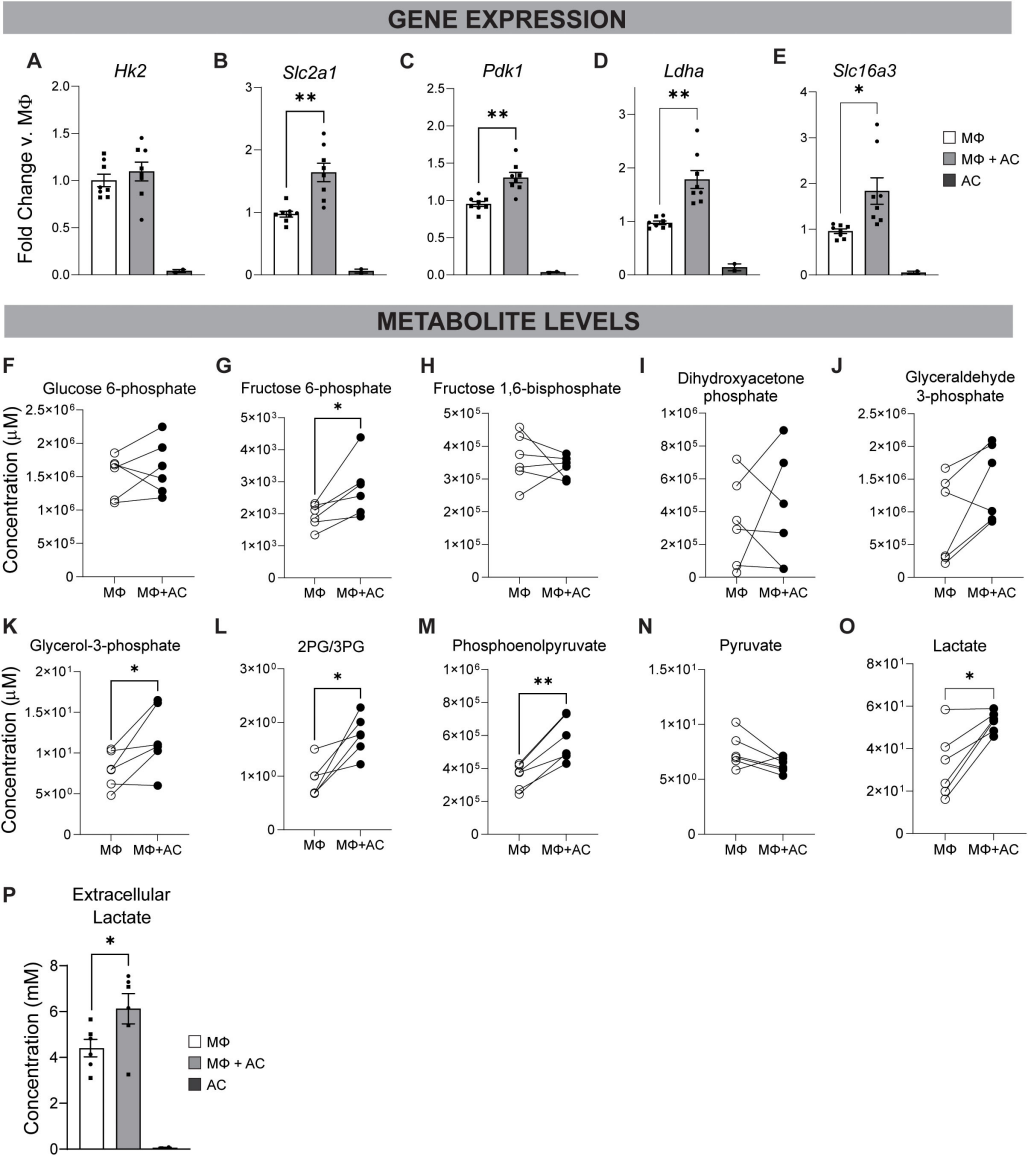
Shift to glycolytic energy metabolism in efferocytic macrophages. MΦ cultured for 18 h alone or with apoptotic primary calvarial osteoblasts (MΦ+AC) were evaluated for glycolysis associated factors. AC- apoptotic calvarial osteoblasts alone. **(A–E)** Gene expression via qRT-PCR. **(F–O)** Intracellular metabolite expression. **(P)** Extracellular lactate expression in culture media. Graphs include data from two independent *in vitro* efferocytosis experiments. Statistical significance evaluated using paired student t-tests: *p < 0.05, **p < 0.01.

The shift in energy metabolism in efferocytic macrophages was further investigated using *in vitro* efferocytosis assays. Macrophages were co-cultured with apoptotic osteoblasts (AC) for 18 h. qRT-PCR analysis revealed that efferocytic macrophages upregulated several genes associated with glycolysis following engulfment of apoptotic osteoblasts. Although *Hk2* (hexokinase 2, an enzyme that converts glucose to glucose-6-phosphate) was not significantly altered (**Figure 5A**), *Slc2a1* (glucose transporter 1, GLUT1), *Pdk1* (pyruvate dehydrogenase kinase 1, inhibits the conversion of pyruvate to Aceytl CoA), *Ldha* (lactate dehydrogenase A, converts pyruvate to lactate), and *Slc16a3* (monocarboxylate transporter 4, MCT4, a lactate/pyruvate transporter) were significantly upregulated in efferocytic macrophages (**Figures 5B–E**). Because PDK1 blocks pyruvate flux into the TCA cycle and LDHA converts pyruvate to lactate, this gene-expression pattern implies increased glucose uptake and enhanced pyruvate-to-lactate conversion in efferocytic macrophages.

Glycolytic shifts beyond the transcriptional level were evaluated using a targeted metabolomics panel (**Figures 5F–O**). Several metabolites, including fructose-6-phosphate, glycerol-3-phosphate, 2 phosphoglycerate (2-PG)/3-PG, phosphoenolpyruvate, and intracellular lactate were significantly elevated in efferocytic macrophages (**Figures 5G, K–M, O**). The observed upregulation of genes associated with lactate production and transport, along with the increase in intracellular lactate, prompted investigation of extracellular lactate levels. A lactate assay performed on the supernatant from control and efferocytic macrophages revealed significantly elevated extracellular lactate in the efferocytic group (**Figure 5P**). Furthermore, lactate levels were negligible in apoptotic cells (AC) alone, confirming that the excess lactate was macrophage-derived as a consequence of metabolic reprogramming.

Although succinate and malate levels were elevated (**Supplementary Figures 5A, B**), other TCA cycle intermediates, including citrate, 2-oxoglutarate, and fumarate, remained unchanged (**Supplementary Figures 5C–E**), suggesting that mitochondrial oxidative phosphorylation is not broadly upregulated during efferocytosis. Instead, the selective accumulation of certain TCA cycle metabolites may reflect a partial engagement of the TCA cycle to maintain cellular redox balance. Together, these findings support a model in which efferocytic macrophages undergo metabolic reprogramming that prioritizes glycolysis, favoring rapid ATP production over reliance on mitochondrial respiration.

### Lactate modulates macrophage polarization and osteoclastogenesis

**Figure 6:**
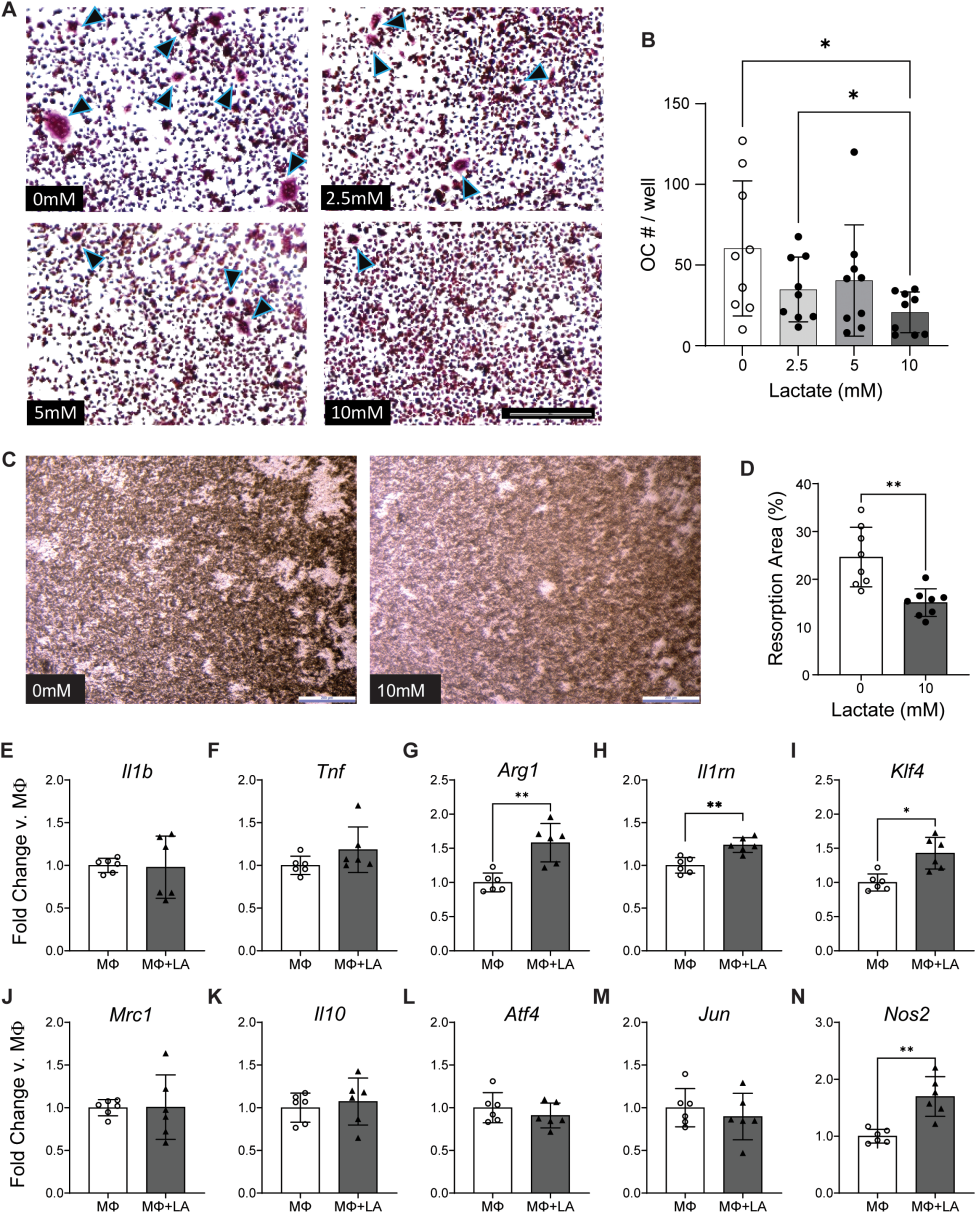
Effect of lactate on key bone cells: osteoclasts, and macrophages. **(A, B)** Representative images and quantification of TRAP staining in osteoclast differentiation with lactate. Statistical significance evaluated using one-way ANOVA: *p < 0.05. **(C, D)** Representative images and quantification of resorption pits after osteoclast differentiation. *p < 0.05; paired t-test. **(E–N)** Gene expression of selected pro-inflammatory, anti-inflammatory, and potential upstream regulators (transcription factors) via qRT-PCR for MΦ cultured with 20 mM extracellular lactate (LA) for 18 h. Statistical significance evaluated using t-test *p < 0.05, **p < 0.01.

Lactate is increasingly recognized not merely as a metabolic byproduct but as an active signaling metabolite that can reprogram macrophages via epigenetic lactylation (32). To determine the effects of lactate on key cells involved in bone homeostasis: osteoblasts, osteoclasts, and macrophages, were investigated. Lactate supplementation of osteogenic media did not significantly alter mineralization levels after 2 weeks in primary bone marrow stromal cells (**Supplementary Figure 6A**) or 3 weeks in primary calvarial OB (**Supplementary Figure 6B**). In contrast, 10 mM lactate supplementation of osteoclast differentiation media significantly reduced the number of multi-nucleated osteoclasts (**Figures 6A, B**) and suppressed resorptive activity as analyzed by reduced pit resorption area (**Figures 6C, D**).

To determine how extracellular lactate affects macrophage phenotype, bone marrow–derived macrophages were cultured with 20 mM lactate. Gene expression determined by qRT-PCR revealed that lactate-treated macrophages did not affect pro-inflammatory factors such as *Il1b* or *Tnf* (**Figures 6E, F**). Conversely, lactate upregulated critical anti-inflammatory factors including *Arg1*, *Il1rn, and Klf4* (**Figures 6G–I**) which are canonical markers and mediators of the alternative (M2-like) macrophage program (33–37). However, *Mrc1* and *Il10* (M2-associated genes) remained unchanged (**Figures 6J, K**). *Atf4* and *Jun*, genes associated with inflammation and bone were also unchanged (**Figures 6L, M**).

Unexpectedly, lactate also increased *Nos2* (iNOS) expression (**Figure 6N**). iNOS produces nitric oxide (NO), a reactive mediator often associated with classically activated (M1-like) macrophages. However, while moderate NO can promote inflammation, high NO is known to inhibit osteoclast differentiation (38). Thus, the lactate-induced rise in *Nos2* (and consequent NO production) may likely contribute to the reduction in osteoclast formation observed. Taken together, our data indicate that lactate can act as an immunometabolite that both reprograms macrophages toward healing modes and suppresses osteoclast-mediated bone resorption.

## Discussion

The role of efferocytosis in maintaining tissue homeostasis and promoting wound healing has been well-documented, and defects in this process have been implicated in various inflammatory pathologies including diabetes and rheumatoid arthritis (2, 3). In studies specific to bone, it has also been shown that impaired efferocytosis impacts bone healing (12–14). Interestingly, we have recently shown that the induction of osteoblast apoptosis resulted in increased efferocytosis near vertebral bone surfaces along with a paradoxical increase in vertebral trabecular bone accrual (15). Despite such findings, the specific contributions of efferocytic macrophages, beyond promoting anti-inflammatory activity in a tissue-specific context, remain poorly defined and are explored in this study.

Most scRNAseq studies investigating macrophages have been conducted in the context of pathological conditions such as cancer (39, 40), rheumatoid arthritis (41), atherosclerosis (42), and periodontitis (43). These studies primarily focused on identifying shifts in macrophage populations under pathological inflammatory conditions but did not specifically investigate changes in macrophage function. Only a few studies to date have examined efferocytosis and the mechanisms driving macrophage reprogramming during this process. Some research groups have conducted in-depth investigations on specific factors differentially expressed during efferocytosis, including *Slc2a1*, which promotes aerobic glycolysis and cytoskeletal remodeling to enable apoptotic cell uptake; *Slc16a1*, involved in lactate release and subsequent anti-inflammatory responses; *Slc7a11*, acting as a brake on aerobic glycolysis; and *Mmp12*, which reduces macrophage migration during efferocytosis (20, 44, 45). Notably, several of these studies emphasized that their findings were specific to efferocytosis and not observed during Fc-mediated phagocytosis. Furthermore, macrophage behavior is heavily influenced by their local environment, and macrophages exhibit distinct phenotypes depending on their tissue of residence (46). Using a congenic parabiosis model, Alonso-Gonzalez and colleagues identified and characterized phagocytic macrophages across various tissues (bone marrow, spleen, intestine, liver, and lung), demonstrating that although unsupervised clustering of their scRNAseq data distinguished phagocytic from non-phagocytic macrophages, the cells clustered with stronger association to their tissue of origin (47).

As our interest is in bone, we focused on bone marrow macrophage efferocytosis of apoptotic osteoblasts and used single-cell RNA sequencing analysis to explore the shifts in macrophage subpopulations during efferocytosis and their subsequent effects relevant to bone. Analysis included unsupervised clustering which revealed two subsets of bone marrow macrophages (clusters 3 and 9) unique to efferocytosis. The distinctive decrease in cluster 0 macrophages during efferocytosis justified its selection as a starting point for pseudotime trajectory analysis. Pathway enrichment and gene expression analyses performed through two independent computational approaches revealed a strong shift toward glycolytic metabolism alongside an increase in anti-inflammatory gene expression in the efferocytosis-associated macrophage subsets. These findings suggest that bone marrow macrophages undergo distinct metabolic and transcriptional reprogramming in response to efferocytosis, with potential implications for the regulation of bone remodeling and inflammation resolution.

The simultaneous glycolytic and anti-inflammatory profile was particularly intriguing, as glycolysis in macrophages has often been associated with “M1-like” or pro-inflammatory activity (16–18), while fatty acid oxidation and oxidative phosphorylation have been associated with “M2-like” or pro-reparative activity (19). Our findings align with emerging evidence that the metabolic process in use alone is insufficient to define a macrophage functional phenotype including the inflammatory wound healing profile. In fact, several genes involved in wound healing and regeneration, such as *Vegfa, Plau, Mif*, and *Cxcr4* were upregulated within the unique c3 and c9 efferocytic macrophages, highlighting potential reprogramming toward enhanced reparative activity.

Further evidence supporting the beneficial role of glycolysis during efferocytosis, reported by the Tabas group, demonstrated that enhanced glycolysis via *Pfkfb2* promoted continual efferocytosis (21) and that efferocytosis-derived lactate increased pro-resolving macrophage proliferation (22). The possibility that intracellular lactate directly influences macrophage gene expression is particularly compelling in light of recent findings that lactate can lactylate histone lysine residues, thereby reprogramming “M1-like” macrophages toward an “M2-like” transcriptional profile (32). In the present study, lactate exposure induced upregulation of *Klf4*, a transcription factor known to cooperate with STAT6 in activating M2-specific genes while repressing pro-inflammatory targets (34). Moreover*, Klf4, Atf4*, and *Jun* were identified as potential upstream regulators associated with the metabolic shift observed in the unique efferocytic macrophage subsets, but these factors may contribute to macrophage reprogramming beyond metabolic control. Collectively, these findings support a model in which lactate produced during efferocytosis facilitates the polarization of macrophages toward an anti-inflammatory phenotype.

Furthermore, lactate secreted by efferocytic macrophages likely impacts the local bone microenvironment. Wu et al. have shown that at early time points 5 mM lactate supplementation can increase *Runx2* (after 24 h), *Alp* (after 24 and 48 h), and *Ocn* (after 48 h) gene expression in MC3T3-E1 cells (48, 49). They also demonstrated increased ALP staining and activity in lactate treated osteoblasts after 96 h (48). Our *in vitro* results did not show any significant difference in mineralized nodule formation of bone marrow stromal cells or primary calvarial osteoblasts when supplemented with lactate for 2 and 3 weeks, respectively. The later endpoints and extended lactate exposure may account for the difference seen in our experiments. When inducing bone marrow cultures to differentiate into osteoclasts, high lactate supplementation significantly suppressed osteoclast formation and reduced their resorptive function. This is concurrent with Seebach et al. who also observed a significant decrease in osteoclast numbers after 6 days, and a significant decrease in osteoclast differentiation-related gene expression (*Nfatc, Acp5, Atp6v0d2)* after 2 days of lactate treatment (50). Further study of the relationship between lactate and gene regulation of osteoclast differentiation will help clarify the underlying mechanism of osteoclast reduction. It is certainly important to consider the temporal nature of efferocytic macrophage-associated lactate and local lactate concentrations in bone, both of which may impact the significance of lactate’s effects on bone *in vivo*.

There is a growing body of evidence suggesting that the glycolytic shift during efferocytosis serves purposes beyond meeting the energy demands of this process. Our study demonstrated that specific subsets of efferocytic macrophages not only exhibited enhanced glycolytic activity but also adopted a more anti-inflammatory and pro-resolving phenotype. We further showed that transcriptional and metabolic changes associated with this glycolytic shift, including increased lactate production, had downstream effects on bone biology. Continued investigation into the drivers of macrophage reprogramming, including transcription factors and metabolites influenced by metabolic reprogramming, may yield valuable insights into the role of efferocytosis and macrophages in tissue-specific contexts. Advancing our understanding of immunometabolism in this setting holds promise for identifying novel therapeutic targets to modulate macrophage function in bone homeostasis, injury, and disease.

## Data Availability

The single cell data discussed in this publication have been deposited in NCBI's Gene Expression Omnibus and are accessible through GEO Series accession number GSE300915 (https://www.ncbi.nlm.nih.gov/geo/query/acc.cgi?acc=GSE300915). Additional inquiries can be directed to the corresponding author.

## Supplementary material

Supplementary Figure 1 Pathway analysis for unique efferocytic cluster 3 & 9. **(A, C)** Summary plot from Advaita iPathwayGuide for **(A)** c3 and **(C)** c9. **(B, D)** Dot plot of top six pathways for **(B)** c3 and **(D)** c9. Statistical significance evaluated using Bonferroni correction. –log(p) > 1.3 (equivalent to p < 0.05) was considered significant.

Supplementary Figure 2 Further characterization of efferocytosis and energy metabolism in single-cell RNA-seq MΦ clusters. Single-cell RNA-sequencing and k-means clustering analysis were performed on pooled control and efferocytic MΦ samples from *in vitro* efferocytosis experiment described in **Figure 1**. **(A)** Heatmap of selected pathways related to (A-C) phagocytosis, (D-M) energy metabolism, and (N-R) miscellaneous macrophage functions for all clusters in the UMAP from **Figure 1B**. Pathways not expressed or with adjusted p > 0.05 are shown in black. **(B)** Heatmap of curated genes involved in efferocytosis for all clusters in the UMAP from **Figure 1B**. **(C)** Heatmaps of curated genes involved in oxidative phosphorylation for clusters along trajectory of interest. Genes not expressed or with adjusted p > 0.05 (Wilcoxon Rank Sum Test) are shown in black.

Supplementary Figure 3 Pathway analysis for gene profiles 1-4. Cells closest to the trajectory of interest identified were used to identify top 1000 genes with a significant relationship to pseudotime. These genes were clustered into five groups based on their expression profile along the trajectory as shown in **Figure 3B**. Summary plots from Advaita iPathwayGuide for gene profile and dot plot of top six pathways for profile 1 **(A, B),** profile 2 **(C, D),** profile 3 **(E, F)** and profile 4 **(G, H)**. Statistical significance evaluated using Bonferroni correction. Dotted red line indicates threshold for significant: –log(p) > 1.3 (equivalent to p < 0.05).

Supplementary Figure 4 Further characterization of inflammation and wound healing in single-cell RNA-seq MΦ clusters. Heatmaps of curated genes involved in **(A)** pro- or anti-inflammatory activity, and **(B)** wound healing (angiogenesis, ECM remodeling, and inflammation) shown for all clusters. Genes not expressed or with adjusted p > 0.05 (Wilcoxon Rank Sum Test) in heatmaps are shown in black.

Supplementary Figure 5 Additional metabolite expression in efferocytic macrophages. MΦ co-cultured for 18 h with apoptotic primary calvarial osteoblasts (MΦ+AC) evaluated for intracellular metabolite expression related to **(A–E)** oxidative phosphorylation. Data from two independent *in vitro* efferocytosis experiments. Statistical significance evaluated using paired student t-tests: *p < 0.05, **p < 0.01.

Supplementary Figure 6 Effect of lactate on osteoblasts. **(A, B)** Representative images and quantification of von Kossa staining in **(A)** 2-week primary BMSC and **(B)** 3-week primary calvarial OB mineralization with lactate. No significant differences (anova) were detected between the groups.
