# Supplementary material for: Efferocytosis-induced metabolic shift in bone macrophages drives lactate production and modulates inflammation and osteoclastogenesis
Source: Front Immunol. 2025 Nov 28;16:1650465. doi: 10.3389/fimmu.2025.1650465 (PMC12698451; doi:10.3389/fimmu.2025.1650465)
Supplement: Supplementary Figure 1 — Pathway analysis for unique efferocytic cluster 3 & 9. (A, C) Summary plot from Advaita iPathwayGuide for (A) c3 and (C) c9. (B, D) Dot plot of top six pathways for (B) c3 and (D) c9. Statistical significance evaluated using Bonferroni correction. –log(p) > 1.3 (equivalent to p < 0.05) was considered significant. [file DataSheet1.pdf]

SUPPLEMENTARY FIGURE 1

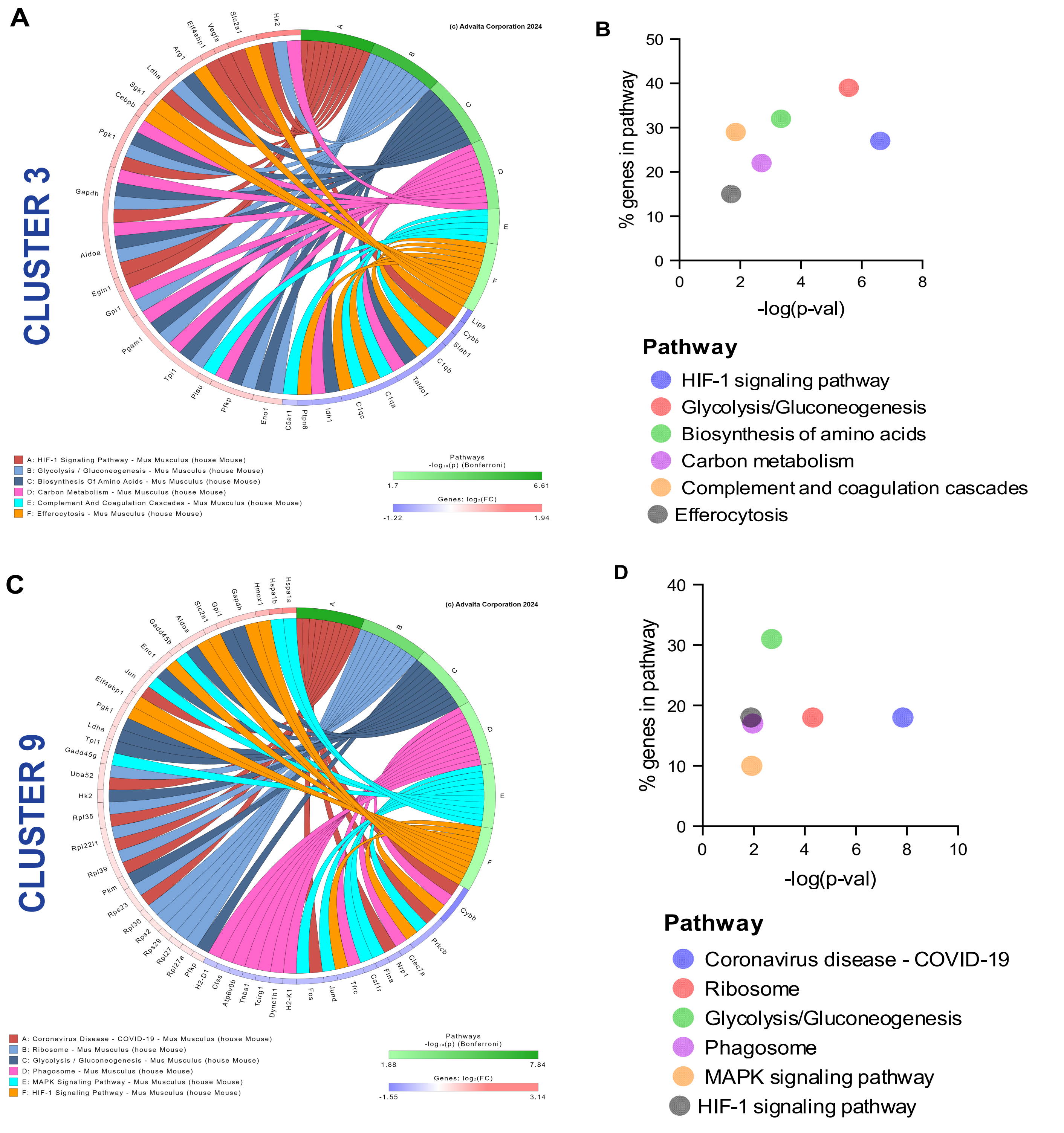

Supplementary Figure 1 | Pathway analysis for unique efferocytic cluster 3 & 9. (A, C) Summary plot from Advaita iPathwayGuide for **(A)** c3 and **(C)** c9. **(B, D)** Dot plot of top six pathways for **(B)** c3 and **(D)** c9. Statistical significance evaluated using Bonferroni correction. log(p) > 1.3 (equivalent to $p < 0.05$) was considered significant.

SUPPLEMENTARY FIGURE 2

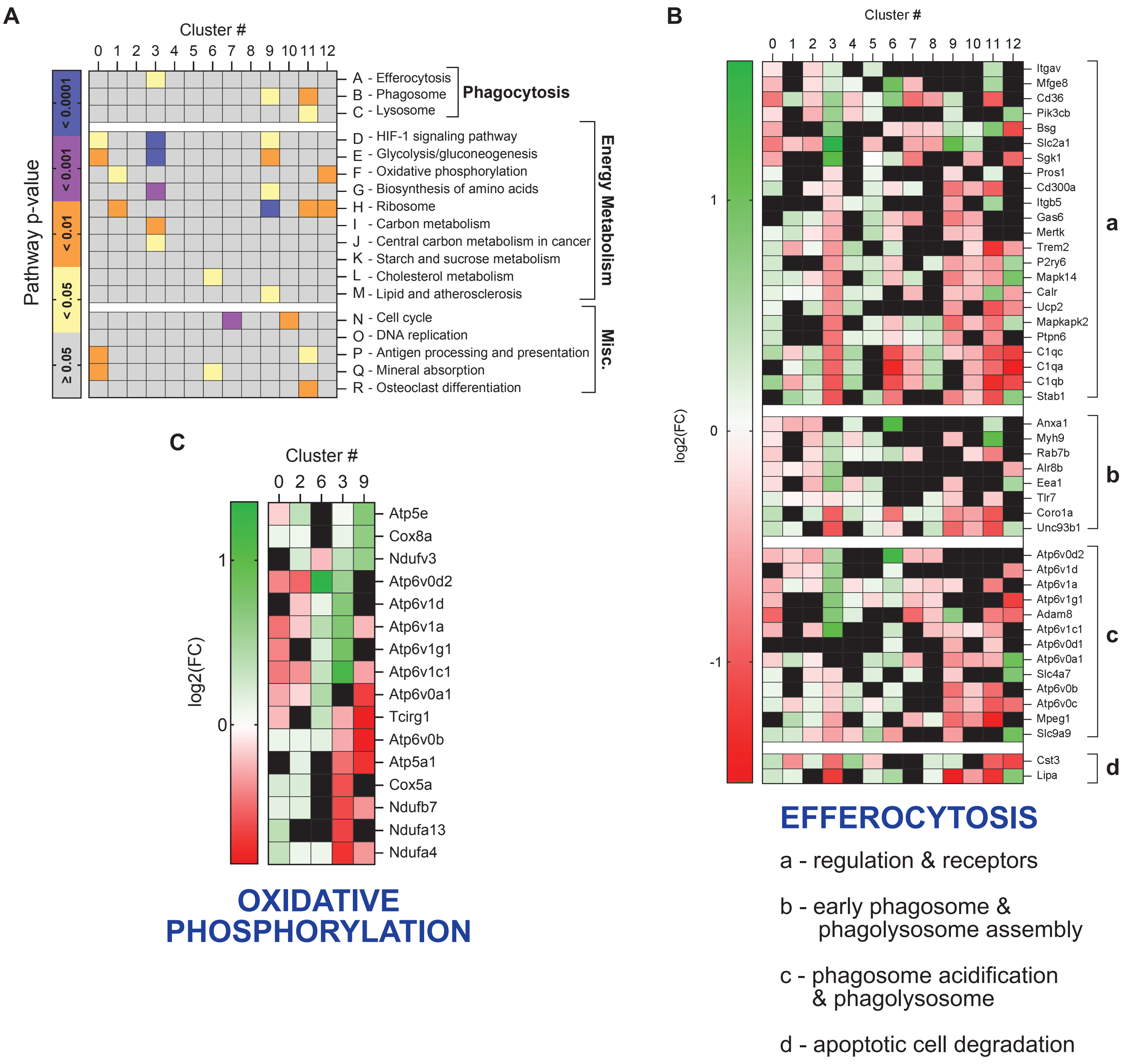

Supplementary Figure 2 | Characterization of efferocytosis and energy metabolism in single-cell RNA-seq MΦ clusters. Single-cell RNA-sequencing and k-means clustering analysis were performed on pooled control and efferocytic MΦ samples from *in vitro* efferocytosis experiment described in Fig 1. **(A)** Heatmap of selected pathways related to (A-C) phagocytosis, (D-M) energy metabolism, and (N-R) miscellaneous macrophage functions for all clusters in the UMAP from Fig 1B. Pathways not expressed or with adjusted p > 0.05 are shown in black. **(B)** Heatmap of curated genes involved in efferocytosis for all clusters in the UMAP from Fig 1B. **(C)** Heatmaps of curated genes involved in oxidative phosphorylation for clusters along trajectory of interest. Genes not expressed or with adjusted p > 0.05 (Wilcoxon Rank Sum Test) are shown in black.

SUPPLEMENTARY FIGURE 3

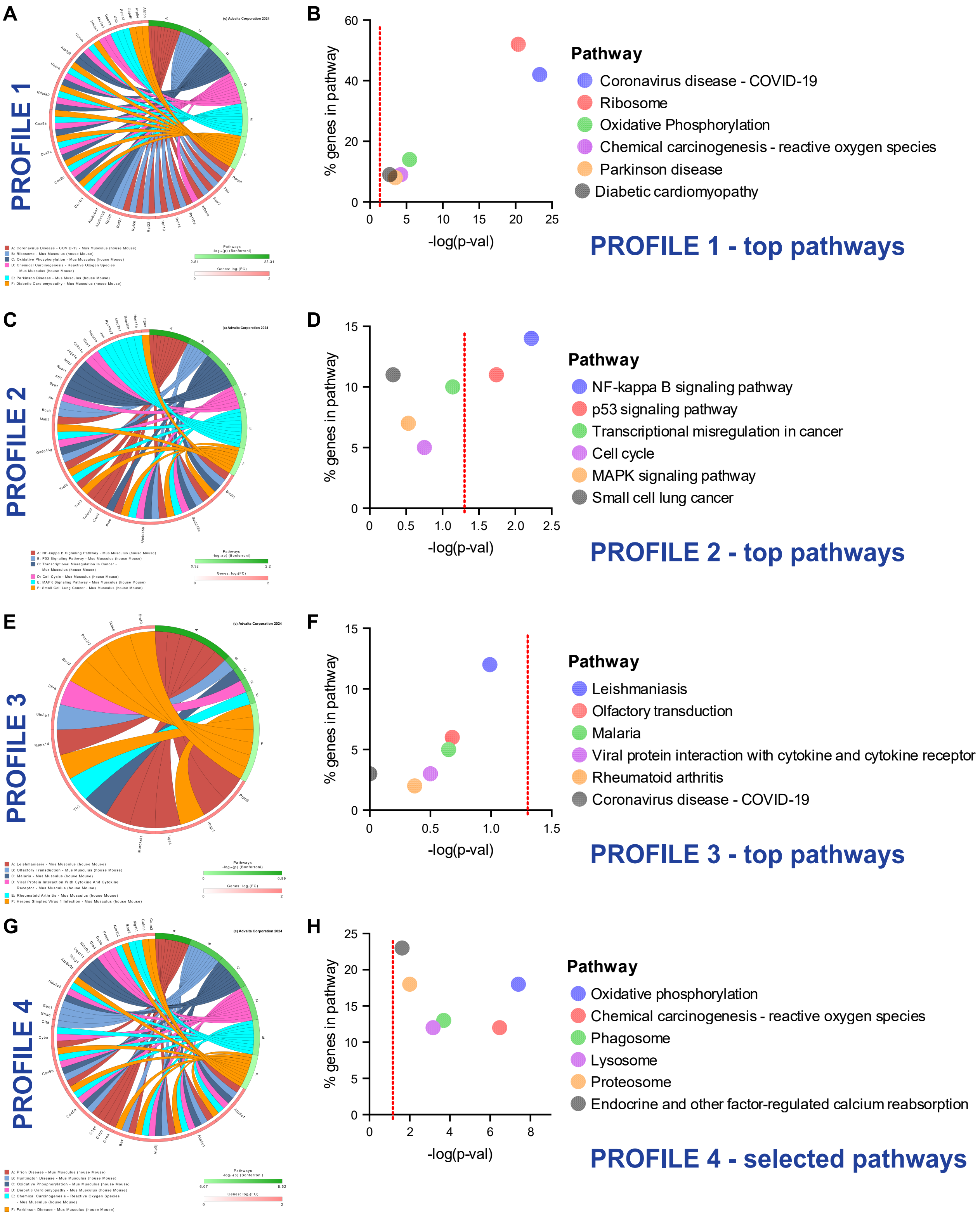

Supplementary Figure 3 | Pathway analysis for gene profiles 1-4. Cells closest to the trajectory of interest identified were used to identify top 1000 genes with a significant relationship to pseudotime. These genes were clustered into five groups based on their expression profile along the trajectory as shown in Fig 3B. Summary plots from Advaita iPathwayGuide for gene profile and dot plot of top six pathways for profile 1 **(A,B),** profile 2 **(C,D),** profile 3 **(E,F)** and profile 4 **(G, H)**. Statistical significance evaluated using Bonferroni correction. Dotted red line indicates threshold for significant: –log(p) > 1.3 (equivalent to $p < 0.05$).

SUPPLEMENTARY FIGURE 4

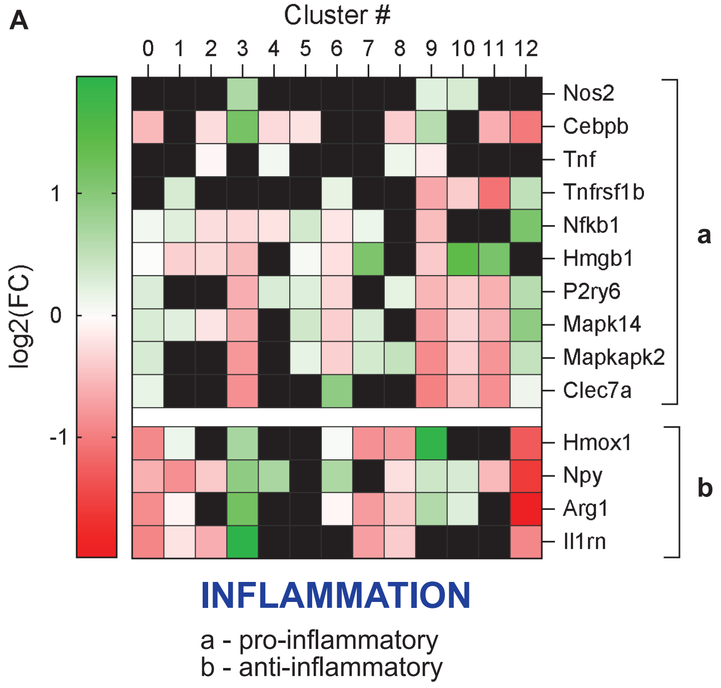

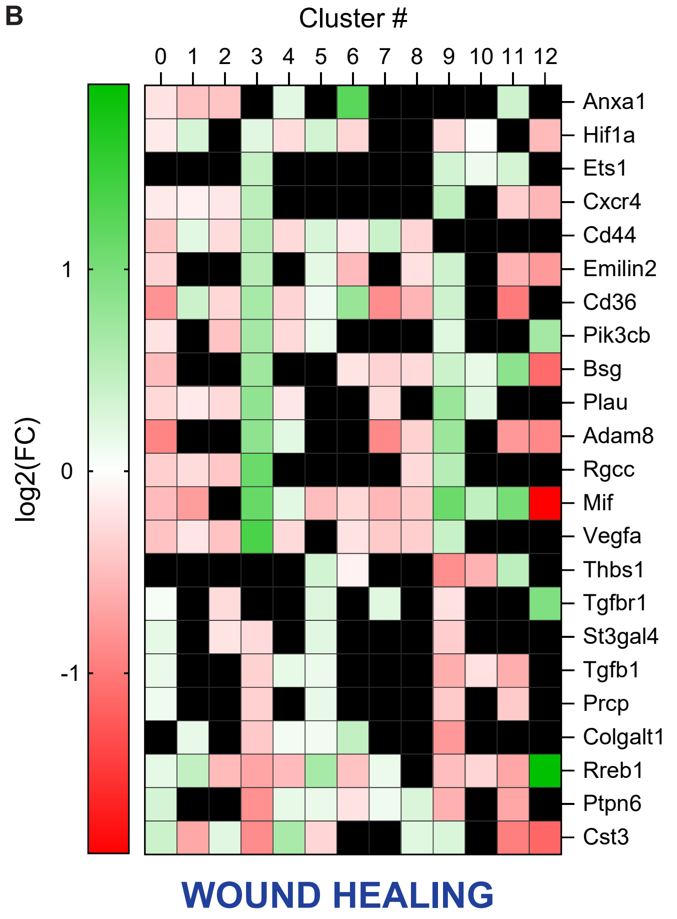

Supplementary Figure 4 | Further characterization of inflammation and wound healing in single-cell RNA-seq MΦ clusters. Heatmaps of curated genes involved in **(A)** pro- or anti-inflammatory activity, and **(B)** wound healing (angiogenesis, ECM remodeling, and inflammation) shown for all clusters. Genes not expressed or with adjusted $p > 0.05$ (Wilcoxon Rank Sum Test) in heatmaps are shown in black.

SUPPLEMENTARY FIGURE 5

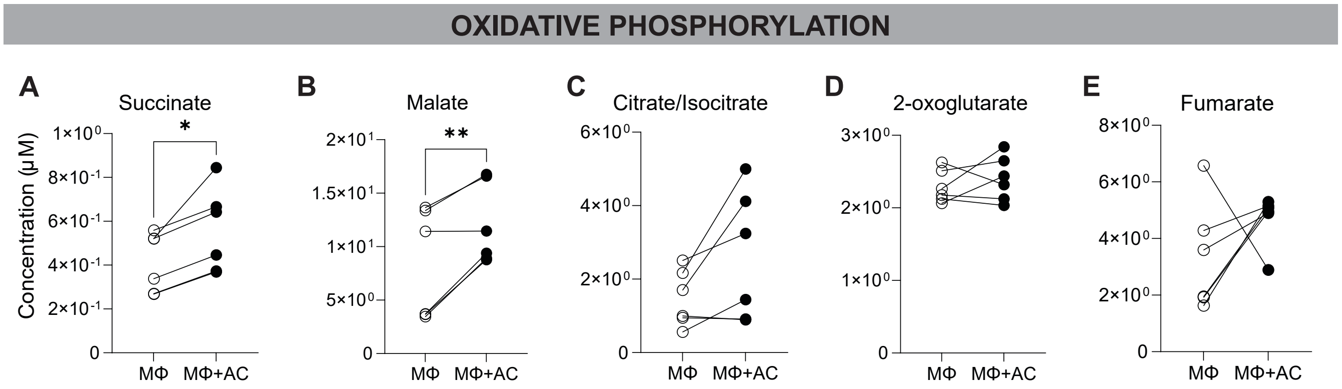

Supplementary Figure 5 | Additional metabolite expression in efferocytic macrophages. MΦ co-cultured for 18 h with apoptotic primary calvarial osteoblasts (MΦ+AC) evaluated for intracellular metabolite expression related to **(A-E)** oxidative phosphorylation. Data from two independent *in vitro* efferocytosis experiments. Statistical significance evaluated using paired student t-tests: *p < 0.05, **p < 0.01**.**

SUPPLEMENTARY FIGURE 6

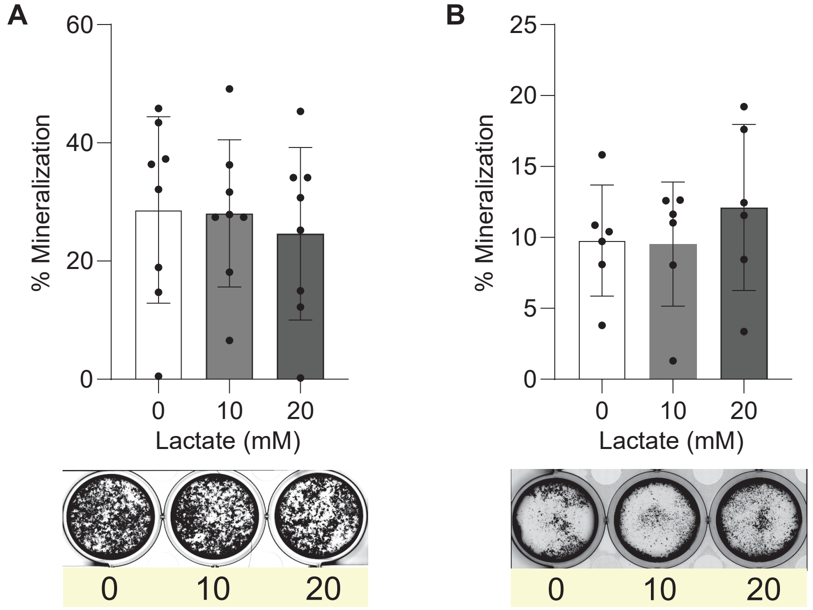

Supplementary Figure 6 | Effect of lactate on osteoblasts. (A-B) Representative images and quantification of von Kossa staining in **(A)** 2-week primary BMSC (n= 8 mice/group) and **(B)** 3-week primary calvarial OB (n= 6 mice/group) mineralization with lactate. Data for A-B is from two independent experiments. No significant differences (ANOVA) were detected between the groups.
